# Cancer-associated fibroblasts in ovarian cancer: research progress

**DOI:** 10.3389/fonc.2025.1504762

**Published:** 2025-03-04

**Authors:** Yuance Xu, Danting Sun, Junqi He, Qin Yao

**Affiliations:** Department of Obstetrics and Gynecology, The Affiliated Hospital of Qingdao University, Qingdao, China

**Keywords:** ovarian cancer, cancer-associated fibroblasts (CAFs), tumor microenvironment, targeted therapy, immune microenvironment

## Abstract

Ovarian cancer, known for its high invasiveness and therapeutic resistance, is one of the leading causes of death from gynecological tumors. The tumor microenvironment (TME) plays a crucial role in the development of ovarian cancer, with cancer-associated fibroblasts (CAFs) being a key non-tumor cell component. They significantly affect the prognosis of ovarian cancer by promoting tumor cell proliferation, invasion, metastasis, immune evasion, and drug resistance. The heterogeneity of CAFs provides a new perspective for targeted therapy in ovarian cancer. This review comprehensively analyzes the mechanisms of action, heterogeneity characteristics, and role in the immune microenvironment of CAFs in ovarian cancer, and discusses targeted therapy strategies for CAFs, aiming to provide new theoretical basis and treatment directions for the treatment of ovarian cancer.

## 1 Research background

Ovarian cancer ranks among the most prevalent malignant neoplasms within the female reproductive system, holding a notable status in terms of incidence and mortality rates among gynecological malignancies (1, 2). The subtle nature of its early indicators often results in a diagnosis at an advanced stage for the majority of patients, characterized by extensive tumor metastasis, thereby presenting significant therapeutic challenges (2). Despite ongoing refinements in surgical procedures and advancements in chemotherapy treatments, the five-year survival rate for ovarian cancer has remained less than optimal, marred by a high recurrence rate and an overall poor prognosis (3). Consequently, there is a critical need to explore the etiology of ovarian cancer and to identify novel therapeutic targets, with the aim of enhancing the survival and quality of life for patients afflicted with this disease.

The tumor microenvironment (TME) encompasses the internal and external milieu in which tumor cells proliferate and evolve, encompassing a complex array of elements including tumor cells, fibroblasts, immune cells, vascular cells, and the extracellular matrix (4, 5). In the context of ovarian cancer development, the TME assumes a pivotal role. Within this ecosystem, cancer-associated fibroblasts (CAFs) emerge as particularly critical cellular constituents. CAFs, which are essentially normal fibroblasts resident in tissues, undergo activation and transformation into CAFs under the influence of signaling molecules released by tumor cells, such as transforming growth factor β (TGF-β) and platelet-derived growth factor (PDGF) (6). These CAFs contribute to the proliferation, invasion, and metastasis of tumor cells by secreting an arsenal of cytokines, chemokines, and factors that remodel the extracellular matrix (7). Concurrently, CAFs suppress the anti-tumor activities of immune cells through the secretion of immunosuppressive factors such as TGF-β, IL-10, and PGE2. These factors inhibit the proliferation and activation of T cells, reducing their ability to kill tumor cells (8). Additionally, CAFs express immune inhibitory molecules like PD-L1, which bind to PD-1 on T cells, further suppressing T cell function (9). The contribution of CAFs to an immunosuppressive tumor microenvironment partially explains the muted response that ovarian cancer patients have to clinically available immunotherapies (10). This highlights the critical need for new therapies targeting CAFs and other components of the TME to enhance the effectiveness of immunotherapy and improve patient outcomes (11).

The heterogeneity of CAFs is a reflection of their functional diversity in the tumor microenvironment (12). Studies have shown that CAFs can be divided into different subpopulations based on their phenotype and function, such as myofibroblast-like CAFs (myCAFs) and inflammatory CAFs (iCAFs) (13). These different CAF subpopulations play different roles in the development of ovarian cancer. For example, myCAFs promote the invasion and metastasis of tumor cells by secreting collagen and fibronectin and other extracellular matrix proteins; iCAFs recruit immunosuppressive cells such as regulatory T cells (Tregs) and myeloid-derived suppressor cells (MDSCs) by secreting IL-6, IL-8 and other inflammatory factors, inhibiting the anti-tumor activity of immune cells (14–16).

With the development of single cell sequencing technology, the heterogeneity of CAFs has been more deeply understood. Researchers have found that the distribution and function of CAFs in ovarian cancer may be closely related to the stage, grade, and prognosis of the tumor. For instance, some subpopulations of CAFs may be related to the early metastasis and recurrence of tumors, while others may be associated with the immune evasion and chemotherapy resistance of tumors. Therefore, in-depth study of the heterogeneity of CAFs is of great clinical significance for revealing the pathogenesis of ovarian cancer, predicting tumor progression and prognosis, and developing personalized treatment plans.

This review aims to comprehensively analyze the mechanisms of action, heterogeneity characteristics, and role in the immune microenvironment of CAFs in ovarian cancer. It will discuss targeted therapy strategies for CAFs, providing new theoretical basis and treatment directions for the treatment of ovarian cancer. The review will cover the following topics: the concept and source of CAFs, their role in the development of ovarian cancer, the impact of CAF heterogeneity on targeted therapy, and the latest preclinical research progress on targeting CAFs for ovarian cancer treatment.

## 2 Concept and source of CAFs

CAFs are different from normal fibroblasts (Normal Fibroblasts, NFs), a type of fibroblast that is in a state of continuous activation, which not only has all the characteristics of NFs but also has more active cell functions, stronger proliferation ability, and higher metabolic status, making it one of the most important cellular components in the TME (11). The characteristics of CAFs are that the cells are slender and spindle-shaped, without the expression of epithelial cells, endothelial cells, and leukocytes and other markers, and lack the cellular characteristics of cancer cells (17).

**Figure 1:**
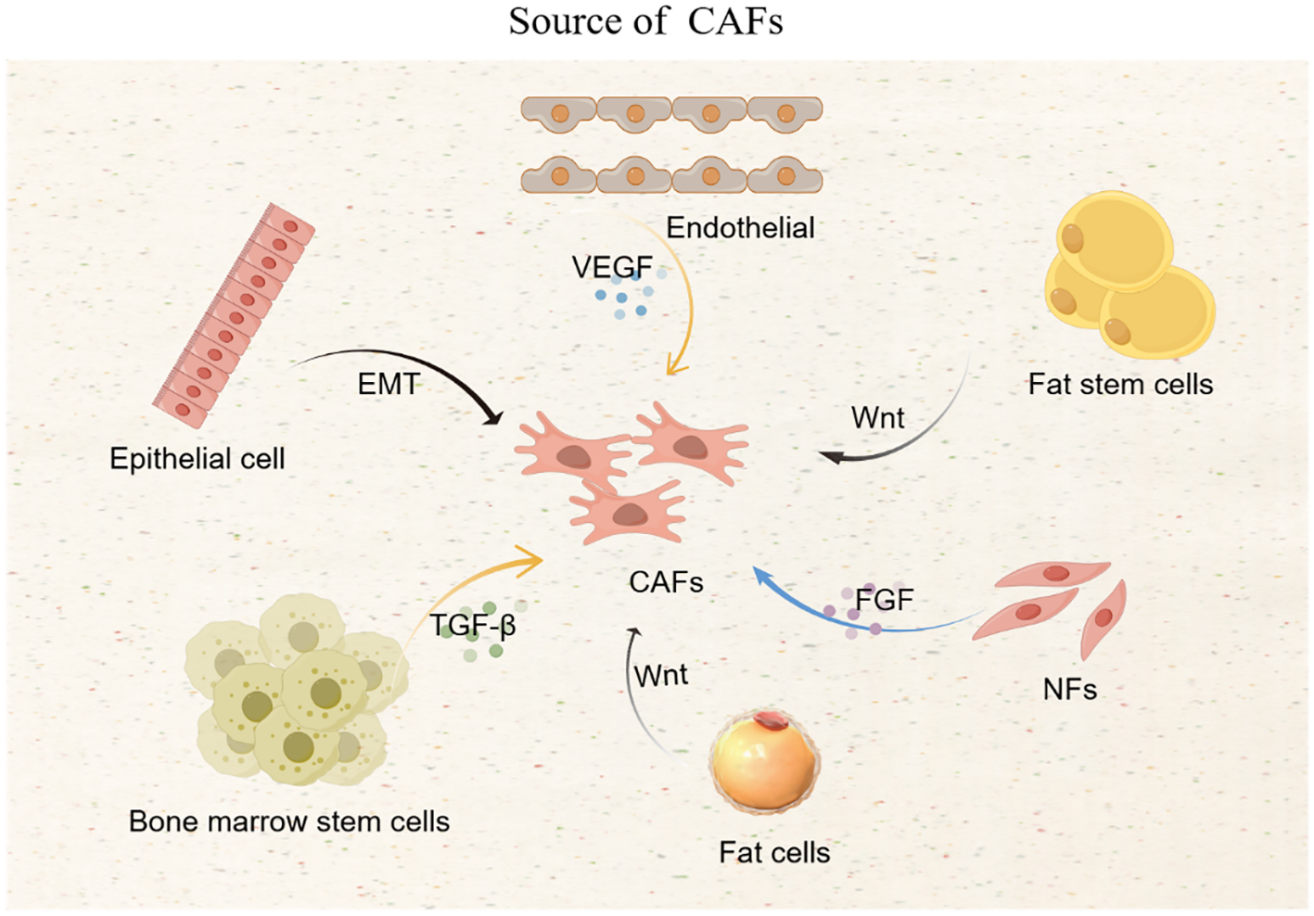
Source of CAFs.

CAFs mainly come from NFs, which are activated by cytokines secreted by cancer cells; epithelial cells and endothelial cells that have undergone epithelial-mesenchymal transition are also important sources of CAFs; bone marrow stem cells, fat stem cells, and pericytes can also be transformed into CAFs. Tumor cells secrete transforming growth factor β (TGF-β), vascular endothelial growth factor (VEGF), fibroblast growth factor (FGF), and white blood cell interleukin (IL) and other cytokines, which promote the transformation of NFs, epithelial cells, endothelial cells, bone marrow stem cells, fat stem cells, and pericytes into CAFs (6). Tumor cells can also transform fat cells and endothelial cells into CAFs through specific signaling pathways, such as the Wnt signaling pathway that prompts fat cells to become CAFs (18) (**Figure 1**).

## 3 The role of CAFs in the development of ovarian cancer

The role of CAFs has been confirmed in various cancers, promoting the occurrence and development of tumors through multiple pathways (19, 20). In the latest research on pancreatic cancer, a new subpopulation of CAFs has been found to exhibit specific anti-tumor effects, indicating the heterogeneity of CAFs (21, 22). Currently, there is relatively little research on CAFs in ovarian cancer, and no special subpopulation of CAFs with anti-tumor effects has been found in ovarian cancer. Most studies have shown that CAFs have the effect of promoting the occurrence and development of ovarian cancer cells, and targeting CAFs is a new treatment method.

### 3.1 CAFs promote the proliferation of ovarian cancer cells

CAFs are the main components of ovarian cancer stromal cells and are in close contact with ovarian cancer cells (OCC). They can exert a promoting effect on tumors through multiple pathways. The interaction between CAFs and OCC promotes glycogenolysis under aerobic conditions and induces the phosphorylation of glycogen metabolic enzymes. Glycogen participates in the glycolysis process, thereby promoting the proliferation ability of OCC (23). CAFs can also promote the angiogenesis or extracellular matrix remodeling of OCC by secreting cytokines and chemokines, causing OCC to proliferate rapidly (24–26). CAFs secrete collagen response medium protein 2 (CRMP2), secretory leukocyte protease inhibitor (SLPI), etc. (27), which can promote the progression of ovarian cancer through specific signaling pathways. CAFs promote the proliferation and growth of OCC by directly participating in cancer metabolism, secreting pro-cancer factors, and regulating signaling pathways.

### 3.2 CAFs promote the invasion and metastasis of ovarian cancer cells

The invasion and metastasis of ovarian cancer refer to the process by which OCC from the primary site, through lymphatic vessels, blood vessels, or the abdominal cavity, reach outside the primary site and continue to grow (28). The high mortality and recurrence rate of ovarian cancer are closely related to its easy invasion and metastasis, and CAFs have the ability to invade the matrix, playing an important role in promoting the invasion and metastasis of ovarian cancer. GLIS family zinc finger 1 (GLIS1) gene can act as a transcriptional activator, inducing the reprogramming of multipotent stem cells into fibroblasts, and plays a role in the differentiation and proliferation of OCC, and is a specific gene overexpressed in CAFs, playing a promoting role in tumors; in addition, the overexpression of the Twist family bHLH transcription factor 1 (Twist1) gene can also promote the transfer of OCC through the interleukin 6 enrichment pathway (29, 30). CAFs secrete the chemokine CXCL14, which can promote the upregulation of long non-coding RNA LINC00092 in OCC, affecting glycolysis and the local support function of CAFs, promoting the transfer of OCC, thus forming a positive feedback loop, which is crucial for the invasion and metastasis of ovarian cancer (30). A variety of long non-coding RNAs (lncRNAs) highly expressed in CAFs, such as CRNDE, MALAT1, MEG3, TP73 -AS1, etc. (31), can promote the invasion and metastasis of ovarian cancer through various signaling pathways. In addition, CAFs release cytokines/chemokines (such as IL-6, IL-8) that inhibit autophagy in OCC, thereby promoting tumor transfer (32). CAFs’ derivative osteomembrane protein (POSTN) promotes the invasion function of OCC by activating the PI3K/Akt regulatory pathway and inducing epithelial-mesenchymal transition (33), and also enhances the promoting effect of CAFs on tumors by integrating through the integrin-mediated NF-κB and TGF-β2 signaling pathways (34). The extracellular vesicles (EV) secreted by OCC can carry miR-630 into NFs, activate the NF-κB pathway, accelerate the activation of CAFs, and promote the transfer of ovarian cancer through a positive feedback loop (35). In addition to indirectly acting on OCC, CAFs can also directly participate in the proliferation of cancer stem cells and the formation of the metastatic niche. In summary, CAFs can promote the occurrence of tumors through specific gene overexpression, secretion of related substances (such as cytokines, chemokines, extracellular vesicles, etc.), mediation of epithelial-mesenchymal transition, and regulation of signaling pathways, and are closely related to the characteristics of invasion and metastasis of ovarian cancer (36).

## 4 Immune evasion by CAFs in ovarian cancer

CAFs play a pivotal role in the immune evasion process of ovarian cancer by modulating the TME to suppress the anti-tumor immune response. They achieve this through several mechanisms. First, CAFs secrete a variety of immunosuppressive cytokines and chemokines, such as TGF-β, IL-10, and PGE2, which inhibit the proliferation and activation of T cells, reducing their ability to recognize and kill tumor cells (8). TGF-β, in particular, is a potent immunosuppressive cytokine that can directly inhibit T cell activation and differentiation, leading to a reduced anti-tumor immune response (9). Second, CAFs express immune checkpoint molecules like PD-L1, which bind to PD-1 on T cells (9), further suppressing T cell function and promoting immune evasion (10). This interaction can lead to T cell exhaustion, a state where T cells become less effective in killing tumor cells (11). Third, CAFs attract and activate regulatory T cells(Tregs) and myeloid suppressor cells(MDSCs) by secreting interleukins(IL-6,IL-8), which can inhibit the antitumor activity of other immune cells (32). Tregs can inhibit the activation of effector T cells, while MDSCs can suppress T cell proliferation and function through the production of reactive oxygen species (ROS) and arginase (31). Finally, CAFs can induce epithelial-mesenchymal transition (EMT) in tumor cells, making them more invasive and less recognizable by the immune system. EMT tumor cells often express lower levels of classical major histocompatibility complex (MHC) molecules, reducing the chance of being recognized by T cells (37). For example, CAFs can secrete TGF-β, which is a key inducer of EMT in ovarian cancer cells (23).The ability of CAFs to facilitate immune evasion has significant implications for the treatment of ovarian cancer. Several strategies are being explored to target CAFs and their secreted factors to enhance the immune response. These include the use of monoclonal antibodies against specific cytokines, small molecules to inhibit signaling pathways, and immunomodulatory agents to reprogram CAFs (38–40). These approaches aim to reduce the immunosuppressive effects of CAFs and improve the overall immune response against ovarian cancer (21).

## 5 Drug resistance mediated by CAFs in ovarian cancer

CAFs contribute to drug resistance in ovarian cancer through multiple mechanisms, which can reduce the sensitivity of tumor cells to chemotherapy and targeted therapies. First, CAFs can activate survival signaling pathways in tumor cells, such as the PI3K/Akt and MAPK pathways, which promote cell survival and resistance to apoptosis induced by chemotherapy drugs (22). For example, the periostin (POSTN) secreted by CAFs can activate the PI3K/Akt signaling pathway, reducing cisplatin-induced apoptosis and leading to drug resistance (41). Second, CAFs can induce epithelial-mesenchymal transition (EMT) in tumor cells, which is associated with increased drug resistance. EMT can lead to changes in cell morphology and behavior, making tumor cells more resistant to chemotherapy (42). The secretion of chemokine CXCL12 by CAFs can activate the CXCR4/Wnt/β-catenin signaling pathway, further reducing the sensitivity to cisplatin (43). Third, CAFs can affect the transport and metabolism of drugs, reducing their availability to tumor cells. For example, the high expression of the lipoma partner gene (LPP) in CAFs can increase the permeability of endothelial cells, reducing the delivery of paclitaxel to cancer cells (44). CAFs can also secrete extracellular vehicles (EVs) carrying miRNAs, such as miR-630, which can target cell cycle proteins like CDKN1A, promoting drug resistance (45–47).

**Figure 2:**
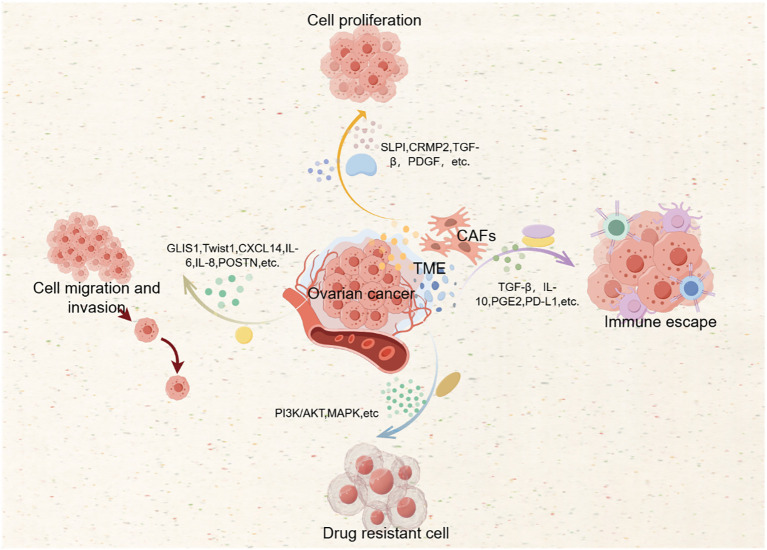
Action mechanism of CAFs.

The role of CAFs in drug resistance has significant clinical implications. Understanding and targeting the mechanisms by which CAFs contribute to drug resistance can help develop more effective treatment strategies for ovarian cancer (44, 46, 48). Potential therapeutic approaches include targeting CAF-secreted factors, which can enhance the sensitivity of tumor cells to chemotherapy. For example, targeting TGF-β signaling can reduce the immunosuppressive effects of CAFs and improve treatment outcomes (2). Reprogramming CAFs to a more quiescent state or to a phenotype that supports anti-tumor immunity can be a promising strategy, achieved through the use of specific small molecules or gene therapy approaches (49). Combining therapies that target CAFs with standard chemotherapy or immunotherapy can enhance the overall treatment efficacy. For example, combining anti-CAFs therapy with immunotherapy can overcome immune evasion and improve patient survival (1). In combination with the action mechanism of CAFs, we drew **Figure 2**.

## 6 The impact of CAF heterogeneity on targeted therapy in ovarian cancer

With the development of emerging gene sequencing technologies, it has been found that CAFs can exhibit different CAF subpopulations with different types and expression levels of markers, and there are great differences in expression among various CAF subpopulations, which is the manifestation of CAF heterogeneity (25). At present, many markers of CAFs have been discovered, such as fibroblast activation protein (FAP), α-smooth muscle actin (α-SMA), and platelet-derived growth factor receptor (PDGF-R) (50). Different CAF subtypes show different biological characteristics. For example, in ovarian cancer, CAFs can promote the invasion and metastasis of OCC by secreting cytokines and chemokines (7); they can also regulate the expression level of immune checkpoint molecules through specific signaling pathways, increasing the immune evasion ability of tumor cells (39). In CAFs, different subpopulations can be divided according to the expression level of markers such as FAP, with high expression of FAP in CAFs inducing tumor cell proliferation, invasion and metastasis, and immune resistance. FAP low-expressing CAFs reduce the promoting effect of FAP high-expressing CAFs on tumor through specific genes such as TCF21 (51); CAFs not only show differences in ovarian cancer but also in other cancers. For example, the four common subtypes of CAFs in breast cancer have different mechanisms of action and expression patterns; even the latest research has found that there are CAF subpopulations that can inhibit the progression of tumor cells in pancreatic cancer (52, 53). It is precisely because of the different expression of different CAF subtypes that the study and analysis of CAF subtypes are one of the current key directions in cancer research.

The heterogeneity of CAFs in ovarian cancer is mainly manifested in aspects of mechanisms of action and expression. The same CAF subpopulation has different phenotypes in different diseases, and even the functions exhibited at different stages of the same tumor are not the same. Due to the diversity of sources and molecular expression differences, CAFs show the heterogeneity of their functions (54, 55). Because CAFs play an important role in the occurrence and development of ovarian cancer, promoting the invasion and metastasis of ovarian cancer and immune evasion, accurately targeting CAFs is of far-reaching significance for the treatment of ovarian cancer (56). Although targeted therapy for CAFs has provided us with new treatment plans for ovarian cancer, due to the heterogeneity of CAF subpopulations, we need a deeper understanding of their mechanisms of action to accurately locate targets in order to treat ovarian cancer (**Table 1**).

**Table 1:** Summary of CAF subtypes in ovarian cancer with references.

Subtype	Marker	Function	Reference
myCAFs	α-SMA, TGF-β	Promote invasion and metastasis	(23, 24)
iCAFs	IL-1, IL-6, TNF-α	Immunosuppression	(31, 32)
FAP-high CAFs	FAP	Tumor promotion	(51)
FAP-low CAFs	FAP	Reduced tumor promotion	(51)

## 7 Preclinical research progress on targeting CAFs for ovarian cancer treatment

CAFs are widely present in the TME of OCC and have the effect of promoting tumor cell proliferation, invasion and metastasis, immune evasion and drug resistance, and their molecular expression is heterogeneous (57, 58). Targeting CAF subpopulations can effectively inhibit tumor progression. Targeting CAFs mainly includes three forms: identifying and killing CAFs through surface markers, interfering with the activation of CAFs, and targeting signaling molecules and regulatory pathways related to CAFs.

At present, a variety of markers have been used to identify CAFs, such as α-smooth muscle actin (α-SMA), platelet-derived growth factor receptor (PDGF-R), and fibroblast activation protein (FAP), etc (59). These markers are highly expressed in CAFs in ovarian cancer stroma, but not detected in normal tissue cells, so CAFs can be killed by identifying surface markers. For example, fibroblast activation protein (FAP) is selectively expressed by CAFs and pericytes in about 90% of human epithelial cancers. Given the high expression and restricted distribution of FAP, targeting the FAP marker can play a role in identifying and killing CAFs, thereby inhibiting the proliferation and development of cancer. Studies have shown that targeting the inhibition of FAP can reduce the recruitment and infiltration of CAFs and is a new method for treating epithelial cancers such as ovarian cancer.

CAFs have a variety of markers on their surface, some of which are also expressed in other stromal cells of ovarian cancer, causing inaccurate identification of CAFs, and bringing difficulties to targeted therapy. Therefore, new targeted treatment plans act on the related expression genes of CAFs, downstream signaling molecules, and regulatory pathways to inhibit the development of ovarian cancer. For example, targeting interleukin 8 (IL-8) (28), chemokine CXCL12 (34), GLIS1 gene (20), lipoma partner gene (LPP) (43), periosteal protein (POSTN), transforming growth factor β (TGF-β) (33), methyltransferase nicotinamide N-methyltransferase (NNMT) (60), human microfiber-related protein 5 (MFAP5) (61), etc. can achieve the purpose of inhibiting the progression of ovarian cancer. Calcitriol can act on the Smad signaling pathway in CAFs, inhibit tumor progression, and extend the patient’s survival time. The monoclonal antibody of human microfiber-related protein 5 can enhance the bioavailability of paclitaxel in ovarian cancer by inhibiting fibrosis and tumor internal microvascular leakage, and inhibit the growth of OCC, which has been applied to clinical treatment (61).

In addition to identifying and killing CAFs, targeting signaling molecules related to CAFs, it is also possible to interfere with the activation of CAFs, that is, to reprogram the function of CAFs. Studies have shown that after the reprogramming of CAFs, it is possible to silence the pro-tumor factors secreted by CAFs while maintaining the overall structure of the extracellular matrix. The use of chitosan nanoparticles (NPs) to deliver the targeted small interfering RNA (siRNA) of human microfiber-related protein 5 for the reprogramming of CAFs will reduce the level of human microfiber-related protein 5 in the TME, thereby inhibiting the metastasis of ovarian cancer (17). It is possible to reprogram CAFs by changing the metabolism inside ovarian cancer. The lysophosphatidic acid (LPA) secreted by OCC stimulates the glycolysis of NFs and CAFs through the hypoxia-inducible factor 1-alpha (HIF1α), and the use of lysophosphatidic acid receptor antagonist (Ki16425) and HIF1α-siRNA can inhibit the glycolysis induced by lysophosphatidic acid, affecting the transformation of NFs into CAFs and inhibiting the progression of ovarian cancer (48). On the other hand, it is also possible to try to transform CAFs back into NFs. In pancreatic ductal adenocarcinoma, CAFs have been successfully transformed into NFs by restoring the level of retinol. At present, there is no effective way to reverse CAFs in ovarian cancer, so trying to transform CAFs into NFs can provide new ideas for the development of new drugs for ovarian cancer (49).

Targeting ovarian cancer CAFs can not only inhibit the proliferation and invasion and metastasis of tumors but also increase the sensitivity of anticancer drugs by changing the tumor microenvironment, reducing the drug resistance and immune evasion of ovarian cancer, and playing an important role in improving the poor prognosis of ovarian cancer, which can be applied to clinical treatment; secondly, by interfering with the activation and reversing CAFs, effective treatment of ovarian cancer can be achieved, which can be used as a starting point for developing therapeutic drugs, making the treatment of ovarian cancer targeting CAFs widely used in clinical practice (**Table 2**).

**Table 2:** Potential CAF targets ovarian cancer.

Potential CAFs targets in ovarian cancer	Identifying and killing CAFs	Interfering the activation of CAFs	Targeting signaling molecules and regulatory pathways
Mode of action	Surface Markers (PDGF-R, FAP, α-SMA)	Metabolic Reprogramming (glycolysis)	Signaling molecules and pathways (IL-8, CXCL12, LPP, GLIS1, POSTN, NNMT, MFAP5, Smad pathways)
Research progress	Preclinical Studies	Preclinical Studies	Preclinical Studies

## 8 Summary

Cancer-associated fibroblasts (CAFs) assume a multifaceted and pivotal role within the tumor microenvironment (TME) of ovarian cancer. They actively foster the proliferation, invasion, and metastasis of tumor cells, while also engaging in immune evasion and the genesis of drug resistance. The heterogeneity of CAFs stems from a diverse array of cell lineages, including normal fibroblasts, epithelial cells, endothelial cells, bone marrow stem cells, adipose stem cells, and pericytes, all of which undergo activation or transformation through a spectrum of distinct signaling pathways. CAFs directly or indirectly affect the progression of ovarian cancer by secreting cytokines, chemokines, and extracellular matrix remodeling factors. In addition, the role of CAFs in immune evasion cannot be ignored. They help tumor cells evade the attack of the immune system by secreting immunosuppressive factors and regulating the function of immune cells. CAFs are also closely related to the drug resistance of tumors, and they may reduce the sensitivity of tumors to chemotherapy drugs by changing drug metabolism or secreting drug pumps, etc.

The future research direction for the treatment of ovarian cancer has been inclined to targeted therapy. Further exploring different subpopulations of CAFs and finding new targets for targeted therapy are key research directions. In addition, targeted therapies for CAFs can be used in combination with other methods to improve efficacy. Finding new immune checkpoint molecules through the process of CAF-induced immune evasion also provides new options for the treatment of ovarian cancer. In summary, whether it is killing CAFs, interfering with the activation of CAFs, or targeting signaling molecules and regulatory pathways related to CAFs, a comprehensive understanding of the properties and mechanisms of action of CAFs is needed to achieve precise positioning. At present, many drugs targeting CAFs have gradually been put into clinical use, but there are still many difficulties that need further study.

Translating preclinical findings into effective clinical therapies is a major challenge. The heterogeneity of ovarian cancer and the variability in patient responses to treatment make it difficult to predict the success of new therapies. Additionally, the invasive nature of ovarian cancer and the high rate of recurrence require more robust and durable treatment strategies. Future research should focus on deeper characterization of CAF subtypes and their specific roles in ovarian cancer. Advanced imaging techniques and single-cell sequencing technologies can provide more detailed insights into the tumor microenvironment. Additionally, combination therapies targeting multiple aspects of the tumor microenvironment, including CAFs, immune cells, and signaling pathways, may offer more effective treatment options.

To sum up, a more profound comprehension of the heterogeneity and functionalities of CAFs is crucial for devising innovative therapeutic strategies. It is imperative that future research endeavors delve deeper into the specific mechanisms by which CAFs operate within ovarian cancer, elucidate the characteristics and roles of various CAFs subpopulations, and investigate their interactions with other cellular elements within the tumor microenvironment. Such inquiries are expected to uncover more potent therapeutic targets, pave the way for the development of novel therapeutic agents, and craft strategies that will enhance the treatment of ovarian cancer, ultimately aiming to bolster the quality of life and clinical outcomes for patients.
